# Sutureless securement of central venous catheters and peripherally inserted central catheters with a novel securement system

**DOI:** 10.1186/2047-2994-4-S1-P214

**Published:** 2015-06-16

**Authors:** K Gregerson, L Rutledge, P Parks

**Affiliations:** 1 Critical and Chronic Care Solutions Division, 3M Company, USA; 2 Exptl Clinical Pharmacol, University of Minnesota, Minneapolis, USA

## Introduction

The use of central venous catheters (CVC) represents a standard of care intrinsic to advanced medical practice worldwide. While the use of these catheters has transformed patient care for the good, adverse events can occur as a result of migration of the catheter and systemic infection. Central venous catheters share the common feature of ending near the atrium but commonly can enter the body through the subclavian and internal jugular veins or can enter through the veins of the arm (PICC). Securement has been shown to be an important factor in reducing the risk of infection. We describe the results of the development of a system of securement that does not require sutures.

## Objectives

1. Analyze strength of securement to the skin/catheter migration using preclinical models.

2. Analyze clinically assessed securement in a spectrum of the continuum of care.

3. Assess skin damage with securement device.

## Methods

Confocal microscopy was used to assess skin damage following attachment and removal of the securement device. Clinical assessment of skin damage using standard dermatological assessment was used as a complement to the confocal microscopy and included measures for erythema, irritation and maceration. Preclinical model testing measured migration of the catheter from the entry site. Migration and catheter loss were assessed clinically.

## Results

Testing for catastrophic failure demonstrated no loss of the system’s ability to maintain system securement. The use of a silicone component to secure the lumens of multi-lumen catheters resulted in little to no skin damage upon removal. Securement was equivalent to existing methods (adhesive anchors and dressings for PICC; sutures for CVC).

## Conclusion

The use of a securement system that includes both dressing and securement device resulted in comparable securement when compared to existing methods (adhesive anchors, sutures, dressings). The use of silicone adhesive as a critical component of attachment to the skin coupled with classical acrylate based adhesive in the remaining portions of the system provided for adequate securement while minimising skin damage.

## Disclosure of interest

K. Gregerson Employee of: 3M Company, L. Rutledge Employee of: 3M Company, P. Parks Employee of: 3M Company.

